# Vitamin D status in Armenian women: a stratified cross-sectional cluster analysis

**DOI:** 10.1038/s41430-021-00934-1

**Published:** 2021-05-13

**Authors:** Nicholas Hutchings, Varta Babalyan, Annemieke C. Heijboer, Sisak Baghdasaryan, Mushegh Qefoyan, Arus Ivanyan, Mariëtte T. Ackermans, Anna Maria Formenti, Olga Lesnyak, Andrea Giustina, John P. Bilezikian

**Affiliations:** 1 School of Medicine, University of California, Irvine, CA USA; 2 Osteoporosis Center of Armenia, Yerevan, Armenia; 3 Endocrine Laboratory, Department of Clinical Chemistry, Amsterdam Gastroenterology Endocrinology Metabolism, Amsterdam UMC, Vrije Universiteit Amsterdam & University of Amsterdam, Amsterdam, The Netherlands; 4 Yerevan State Medical University, Yerevan, Armenia; 5 Institute of Endocrine and Metabolic Sciences, San Raffaele, IRCCS Hospital, Vita-Salute University, Milano, Italy; 6 North West State Medical University named after I.I. Mechnikov, Saint Petersburg, Russia; 7 College of Physicians and Surgeons, Columbia University, New York, NY USA

**Keywords:** Osteoporosis, Nutrition

## Abstract

**Background:**

Vitamin D plays a critical role in skeletal development and maintenance, thus sufficiency is an important goal for public health programs. Given the absence of foods fortified in vitamin D in Armenia, we hypothesized that vitamin D insufficiency would be widespread.

**Methods:**

We conducted a random modified cluster model survey of vitamin D status of women in the country. We measured 25-hydroxyvitamin D [25(OH)D] by liquid chromatography mass spectrometry in dried blood spot samples and utilized a questionnaire to assess lifestyle factors.

**Results:**

In summer, we sampled 1206 participants from 40 communities in Armenia. Mean 25-OH D level among women aged 18–24 was 20 ± 8 ng/mL; aged 25–64 was 21 ± 7 ng/mL; and >65 was 18 ± 8 ng/mL. The country-wide mean of the entire female population was 20 ± 8 ng/mL. A majority (>54%) had 25-OH D levels <20 ng/mL with nearly 13% having 25-OH D levels <12 ng/mL. Participants who reported calcium, vitamin D, or multivitamin supplementation had higher levels of 25-OH D (*p* values 0.004, 0.0002, and 0.03 respectively) as did pre- vs. postmenopausal women (*p* = 0.01), pregnant vs. nonpregnant women (*p* = <0.0001), and women who had experienced a sunburn in the past year (*p* = 0.004).

**Conclusion:**

In Armenia, there is a high prevalence of vitamin D insufficiency. This information provides data that can be used to inform public health directives to address this pervasive threat to optimal health.

## Introduction

Vitamin D plays a critical role in human health and development throughout life. Childhood deficiency of vitamin D leads to rickets. Vitamin D deficiency in adult life can be associated with osteomalacia, lower bone mineral density, frailty, and falls predisposing to osteoporotic fragility fracture [1]. Inadequate gastrointestinal calcium absorption and defective mineralization of the skeleton due to vitamin D deficiency both contribute to these skeletal disorders which are associated with increased fracture risk [2]. In addition, vitamin D has been implicated in a plethora of other biological processes, such as innate and adaptive immunity. Bacterial and viral infections, cancers, cardiovascular disease, diabetes mellitus, rheumatoid arthritis, and multiple sclerosis have all been linked to vitamin D insufficiency although the evidence for a direct or conclusive role in these nonskeletal systems is far from conclusive [3–12]. Nevertheless, it is universally agreed that appropriate vitamin D status is a critical goal both for clinicians treating patients on an individual level, and for public health programs seeking to improve the health of populations. Unfortunately, deficiency is widespread, with an estimated 1 billion people worldwide deficient in vitamin D [3, 13]. In the Middle East and Northern China, for example, up to 50% of the population has levels below 12 ng/mL [14].

Natural sources of vitamin D include sun exposure and some foods. Other than fatty fish and corn products, however, few natural foods contain substantial quantities of the vitamin. In addition, limited direct skin exposure, widely recommended to reduce the risk of skin cancer, renders the sun a rather minor source of vitamin D [15, 16]. Circulating vitamin D is routinely measured in its 25-hydroxylated form. While not the biologically active form of vitamin D, 25-hydroxyvitamin D (25-OH D) is an accurate index of body stores [17]. While there is some controversy over what is the desired level of 25-OH D to ensure vitamin D sufficiency with some in the literature indicating >30 ng/mL as the optimal level [18], most experts believe that levels >20 ng/mL (50 nmol/L) are the lowest compatible with optimized vitamin D status. From a public health point of view, however, if the population mean is 20 ng/mL, 50% of the population would be below this minimal level of vitamin D sufficiency. Levels below 20 ng/mL are defined as insufficient with levels below 12 ng/mL frankly deficient [19].

Armenia is a small middle-income country in the south Caucasus, with a population of just around three million spread over nearly 30,000 square kilometers [20]. The mountainous country has a great diversity of climates based on altitude, but the capital city where a plurality of the population resides is classified as semidesert and dry steppe, with an average of 2700 h of of sun per year [21]. Osteoporosis is an emerging clinical and social problem in Armenia against which great strides have been made in recent years in the country toward improving diagnosis and treatment [22]. However, any effort to prevent and treat bone fragility begins by ensuring that patients are adequate in their vitamin D status [23, 24]. Unfortunately, in Armenia, as in many South European countries [25], food fortification with vitamin D has never been undertaken, and this is likely to predispose those in Armenia to vitamin D insufficiency. Indeed, initial studies of small convenience samples of older women in Yerevan, the capital of Armenia, have discovered levels that were frankly very low, under 10 ng/mL [26].

The aim of our study was to determine the prevalence of vitamin D insufficiency among women in Armenia, and to identify subgroups of the population that are most at risk for the consequences of deficiency in vitamin D. Such data could be used to design and implement educational and interventional programs to correct the problem.

## Methods

We conducted a cross-sectional cluster model study to measure levels of 25-OH D from a representative sample of women in Armenia.

### Participant selection

We aimed to recruit 1232 participants, enough to identify a prevalence of insufficiency of 50% with a confidence interval of 95% and ±5% precision, with an estimated cluster model design effect of 2 [27, 28]. Furthermore, the estimate is sufficient to allow comparison between the three age strata of young women (aged 18–24), middle-age women (aged 25–64), and older women (65 or older), with a significance level *α* of 0.05 and power (1 − *β*) of 80%, assuming normal distribution of values and expecting a difference of at least 5 ngl/mL difference in the mean values between the groups, with an expected population standard deviation of 6.8 ng/mL based on prior studies in other populations [29–31].

All citizens of Armenia are registered in a public polyclinic. We obtained a list of all polyclinics and the size of the population registered at that clinic. We then created a list where each polyclinic appears *n* times, where *n* = ^registered population^/_100_ in order to weight each clinic by population. We then divided the total number of list entries by 40 to give a number *y* and randomly selected a number *z* from 0 to *y* using the random-number generator function in Excel. We selected the *z*^th^ entry from the list, the (*z* + *y*)^th^ entry, the (*z* + 2*y*)^th^ entry, and so-on until 40 polyclinics had been selected from the list. At each polyclinic, we obtained the list of women registered at the clinic aged 18–24, and following a similar process as above, we divided the total number registrants by 10 to give number *y*, randomly selected a number *z* from 0 to *y*, and select the *z*^th^ entry, the (*z* + 2*y*)^th^ entry, and so-on until 10 women had been selected from the list. This process was completed four times, providing lists *A*, *B*, *C*, and *D*. The individuals from list *A* were then contacted via telephone by members of the staff of the polyclinic and were invited to participate in the study. If any were unreachable or declined to participate, an individual from list *B* was contacted, and then from *C* if necessary, and so on, until ten women were recruited. This process was then repeated for women between the ages of 25–64 to select 15 participants, and for women age 65 or over for 10 participants, such that 35 women total were recruited for each site.

Inclusion criteria were appropriate age and permanent residence in the community. Exclusion criteria are having taken vitamin D supplements of any time in the past month and being unable to provide informed consent.

### Acquisition of blood sample and other data

At each site, following written informed consent, participants were provided with a 38-item multiple choice and short answer questionnaire about demographics, pertinent medical history, sun exposure, activity, and diet, which they completed and returned to the study staff. They then underwent measurement of height and weight.

Blood was obtained from all participants by finger prick, the first drop of which was wiped by clean cotton gauze and discarded. The second drop of blood was pipetted on to the testing strip and read by the hand-held digital point-of-care Mission Hb device to measure hematocrit. A further five drops of blood were expressed and spotted onto a Whatman 903 dried blood spot (DBS) card [32]. The card was then set to dry for 2 h at room temperature, placed in a sealable plastic bag with silica to maintain low humidity, and stored in a freezer until all samples were obtained.

### Laboratory analyses

All DBS cards were shipped to Amsterdam, to the endocrine laboratory of the Amsterdam UMC for analysis of 25-OH D via liquid chromatography mass spectrometry (LC-MS/MS). In short, four 1/8 inch blood spots were punched out of each DBS sample and 50 µL H_2_O was added. After equilibration and mixing, internal standards ^13^C_5_-25-OH D_3_ (IsoSciences, Ambler, PA, USA) and ^3^H_6_-25-OH D_2_ (TRC, North York, Canada) diluted in acetonitrile were added. After sonification, supernatant was pipetted into a 96 well plate, dried under N_2_ at 35 °C, and re-dissolved in methanol/H_2_O (50/50 v/v). Samples were analyzed using ID-2D-LC-MS/MS (Acquity-Xevo TQS, Waters Corp., Milford, MA). The lower limit of quantitation was set at 2 ng/mL for both 25-OH D_3_ and 25-OH D_2_. The intra-assay variation was ≤6% for 25-OH D_3_ and ≤8% for 25-OH D_2_ over the whole concentration range. The inter-assay variation was ≤8% and ≤9% for 25-OH D_3_ and 25-OH D_2_ respectively over the whole concentration range. 25-OH D_2_ and 25-OH D_3_ were measured separately, but in this study, we used the sum of both. This method shows a strong correlation between DBS and plasma-obtained measurements of 25-OH D (*R* = 0.98) (internal quality control data from the laboratory of the co-author, ACH). Based on this correlation, results were calibrated against plasma measurements to allow for clinical interpretation. The plasma 25-OH D method used for the calibration is standardized well, as published previously [33].

### Statistical analyses

25-OH D levels were used to produce summary statistics for the country as a whole, and for each age stratum. Comparisons between age strata were conducted via *t* tests, investigation of correlations between questionnaire responses and vitamin D level was conducted via *t* test for dichotomous variables, ANOVA for non-dichotomous categorical variables, and logistic regression for continuous variables. All statistical calculations were conducted using Excel (Microsoft, Redmond, WA).

## Results

Between May and July of 2019, samples were obtained by the DBS method from 1238 participants in 40 clinics. 32 (2.5%) of the total could not be utilized for insufficient sample size, duplicate sample, or a missing questionnaire. A total of 1206 participants were included in the final analysis. All ten provinces and Yerevan, the capital city of Armenia, were included, spanning lattitudes 39°26′ to 41°08′ north. The distribution by age is given here: 339 women aged 18–24 (mean age 21 ± 3.5 years), 522 women aged 25–64 (mean age 46 ± 11.7 years), and 345 women aged 65 and older (mean age 71 ± 5.5 years). See Table 1.

**Table 1:** Demographics of participants.

	Women aged 18–24	Women aged 25–64	Women aged 64+
Count	339	522	345
Mean age (SD)	21 (3.5) years	46 (11.7) years	71 (5.5) years
Mean weight	57 (13) kg	73 (19) kg	74 (16) kg

Only five samples showed a 25-OH D_2_ concentration above the lower limit of quantification. Four samples showed a 25-OH D_2_ concentration of 2.8 ng/mL and one sample a concentration of 4.8 ng/mL. Thus, the measured 25-OH D level for the vast majority of subjects in this study reflected the 25-OH D_3_ form of the vitamin.

Mean 25-OH D level of the entire population was 20 ± 8 ng/mL. A majority of the population (>54%) had 25-OH D levels <20 ng/mL with nearly 13% in the frankly deficient range of <12 ng/mL.

**Fig. 1:**
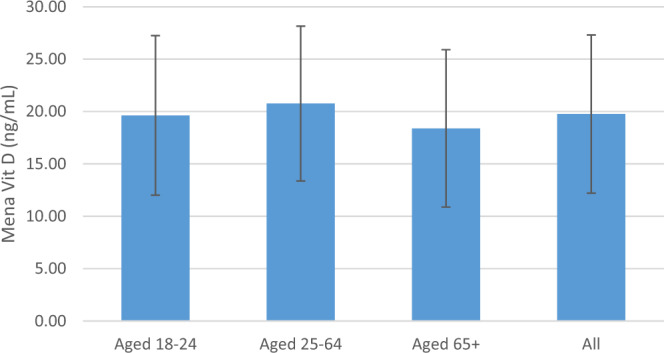
Mean vitamin D level by age stratum. Mean level of vitamin D in ng/mL for each of the three age strata and for the entire study population, with error bars indicating plus/minus standard deviation.

**Fig. 2:**
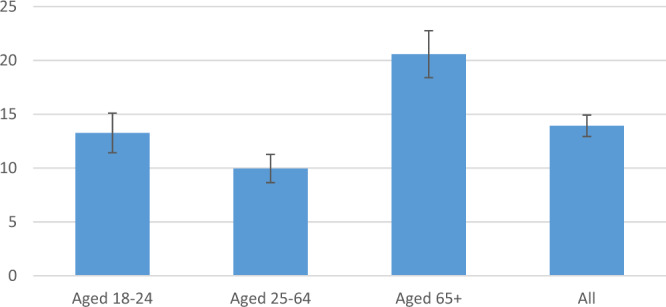
Percentage of participants with vitamin D level below 12 ng/mL. Percentage of participants with vitain D level below 12 ng/mL, the level of deficiency, for each of the three age stratum and for the entire study population, with error bars indicating plus/minus standard error of measurement.

The mean 25-OH D level among women aged 18–24 was 20 ± 8 ng/mL with 13% with levels below 12 ng/mL; among women aged 25–64 was 21 ± 7 ng/mL with 10% with levels below 12 ng/mL; and among women >65 was 18 ng/mL (SD 7.5) a full fifth of the population (21%) with levels below 12 ng/mL. There was no significant difference in mean 25-OH D level between the age strata. (Figs. 1, 2). Mean 25-OH D level was slightly lower among urban dwellers in the capital city of Yerevan compared to other sites (19 vs. 20 ng/mL, *p* = 0.00004).

Participants who reported calcium, vitamin D, or multivitamin supplementation had statistically higher levels of 25-OH D (*p* values 0.004, 0.0002, and 0.03 respectively) as did pre- vs. postmenopausal women (*p* = 0.01), pregnant vs. nonpregnant women (*p* = <0.0001) and women who had experienced a sunburn in the past year (*p* = 0.004). There was no statistically significant relationship between the 25-OH D level and reported smoking, alcohol use, sun exposure, sun protection, tanning, physical activity, or dietary intake of foods rich in vitamin D, however somewhat unexpectedly, sitting more than 3 h a day was associated with higher levels of 25-OH D. See Table 2.

**Table 2:** Mean vitamin D levels by survey response.

	Count of respondents who replied affirmatively (% of total respondents)^a^	Mean 25-OH D of respondents who answered affirmatively	Mean 25-OH D of respondents who answered negatively	*p* value, calculated via *T* test
Currently ill	579 (50%)	20 ng/mL	20 ng/mL	0.4
Chronic disease	374 (34%)	20 ng/mL	19 ng/mL	0.3
Current medication use	631 (54%)	20 ng/mL	20 ng/mL	1
Calcium supplements	164 (14%)	21 ng/mL	20 ng/mL	0.004
Vitamin D supplements	111 (9.5%)	23 ng/mL	19 ng/mL	<0.0001
Multivitamin supplement	48 (4.2%)	22 ng/mL	20 ng/mL	0.03
Number of children	–	–	–	0.09^b^
Premenopause	621 (51%)	20 ng/mL	19 ng/mL	0.01
Early menopause^c^	182 (33%)	19 ng/mL	20 ng/mL	0.7
Currently pregnant	37 (3.1%)	28 ng/mL	20 ng/mL	<0.0001
Currently breast feeding	44 (3.7%)	20 ng/mL	20 ng/mL	0.9
Previous fracture	230 (20%)	19 ng/mL	20 ng/mL	0.4
Parent broken hip	67 (5.8%)	20 ng/mL	20 ng/mL	0.8
Smoking	17 (1.4%)	22 ng/mL	20 ng/mL	0.2
Alcohol use	12 (1.0%)	19 ng/mL	20 ng/mL	0.8
Sunburn in past year	171 (16%)	21 ng/mL	19 ng/mL	0.004
Vigorous activity	219 (19%)	20 ng/mL	20 ng/mL	0.2
Moderate activity	250 (25%)	20 ng/mL	20 ng/mL	0.4
Walking or biking	473 (44%)	20 ng/mL	20 ng/mL	0.4
Vigorous sport	108 (10%)	21 ng/mL	20 ng/mL	0.2
Moderate sport	125 (12%)	20 ng/mL	20 ng/mL	0.6
Sitting more than 3 h per day	467 (52%)	22 ng/mL	21 ng/mL	<0.0001
Yerevan dwelling	417 (35%)	19 ng/mL	20 ng/mL	<0.0001

^a^Not all respondents answered every question, thus the percentage is of those who responded, not of the total study population.

^b^Calculated via ANOVA.

^c^Menopause before age 45.

## Discussion

Vitamin D insufficiency is widespread throughout the world [7, 34]. This report further documents this statement. The results of our study show a strikingly high prevalence of vitamin D deficiency (<12/ng/mL) among women in Armenia with 13% nationwide and 20% of those over the age of 65, despite the fact that Armenia’s climate brings mild and sunny weather. These data are even more striking since it is known that vitamin D levels do dramatically fall during the winter and likely largely underestimate the real prevalence and severity of hypovitaminosis D during the year. There were differences in the mean levels between the age strata (18–24 years old, 25–64 years old, and 65 and older), as well as with other reported demographic and lifestyle characteristics, however they are too small to be of clinical relevance. Indeed, the small difference across the entire population with all subgroups being quite similar to each other substantiates the conclusion that vitamin D insufficiency is widespread in Armenia. The one notable exception is that of pregnant women: although a small percentage of our study population, their mean vitamin D was markedly higher than the population mean, likely secondary to the widespread use of vitamin supplementation during pregnancy which demonstrates the importance and impact of prenatal care in this population.

Armenia has a young population, with nearly 20% under the age of 15, and 53% under the age of 35 [35]. While we did not include children or men in this study, it can be reasonably assumed that the dietary intake and lifestyle of children and men are sufficiently similar to that of women to allow us to extrapolate these study results those populations as well. Thus, an estimated rate of frank vitamin D deficiency with levels below 12 ng/mL of 10–15% in children and in women of reproductive age is very concerning given the risk of osteomalacia and rickets in infancy and childhood.

Public health interventions designed to address endemic vitamin D insufficiency have largely focused on fortification of foods. The United States and Canada have fortified dairy products since the 1940s, but recent research has investigated alternative carriers such as vegetable oil, flour, and fruit juice. Biofortification by supplementing livestock feed with vitamin D has shown promise in increasing bioavailable vitamin D in eggs and meat products. Such interventions, taken place on a national level, have consistently shown improvement in population mean vitamin D level and decrease in childhood rickets [36]. Recent research findings of improvements in bone mineral density with achievement of vitamin D sufficiency in smaller trials suggest that this benefit could be replicated on the population level as well [37]. Given the high rate of vitamin D insufficiency in Armenia, there is a clear need for public health intervention, necessarily tailored to the cultural and dietary characteristics of the community, for which the methods and options are many, and the impact very clear.

Major strengths of the study include the method of recruitment, utilizing existing public health network infrastructure in a random and representative fashion. In addition, the use of DBS cards for sample collection provided an easy, reliable, and stable method to obtain, store, and analyse blood samples for vitamin D. Moreover, the accurate and reliable method for quantification of 25-OH D, a well standardized ID-LC-MS/MS method, was a strength. A further strength was the large sample size (approximately one in every 1300 women in Armenia participated in the study), short time period of collection (over 3 months in one summer), and concurrent collection of comprehensive and applicable demographic and other characteristics. Limitations of the study include those inherent to the cluster design model, that is, it may not be perfectly representative of the population, especially with regard to those living in smaller rural communities, and the use of self-reporting for the questionnaire which has the classic drawbacks of poor recall, haste or question fatigue, and simple unreliability in participant self-reporting. Nevertheless, this study provides much-needed data to inform and bolster public health directives to address this pervasive threat to optimal skeletal health.
